# High-density Grid and Magnetic Resonance Imaging Working Together

**DOI:** 10.19102/icrm.2021.120113S

**Published:** 2021-01-15

**Authors:** Gabriel Martín, Sandra Cabrera, Oriol Martín Tejada, Amin Elamrani

**Affiliations:** ^1^Hospital Universitario de Tarragona Joan XXIII, Tarragona, Spain

**Keywords:** Ischemic heart disease, ventricular tachycardia, voltage substrate mapping

A 65-year-old man with arterial hypertension, dyslipidemia, and ischemic cardiomyopathy together with three-vessel coronary disease, for which surgical revascularization was performed in 1999, presented to the clinic. In the acute postoperative period, he had experienced a cerebrovascular accident with the sequelae of left hemiplegia, then an episode of cardiorespiratory arrest in June 2019, with an echocardiogram showing dilated ischemic cardiomyopathy with severe ventricular dysfunction, so a single-chamber automatic defibrillator was implanted. He was discharged with bisoprolol and amiodarone as antiarrhythmic treatment. In October 2020, he displayed episodes of sustained ventricular tachycardia (VT) treated with electrical cardioversion. His bisoprolol and amiodarone were exchanged for sotalol. Despite this change, however, the patient had new episodes of VT requiring implantable cardioverter-defibrillators shocks, so ablation was decided. Cardiac magnetic resonance imaging showed extensive areas of fibrosis in the basal and middle areas of the inferoseptal, inferolateral, and anterolateral segments of the left ventricle.

In November 2020, we performed mapping and ablation using a double-access (transseptal and retroaortic) approach. We used the Advisor™ HD Grid Mapping Catheter, Sensor Enabled™ for mapping and TactiCath™ SE catheter for ablation. Initially, we performed substrate mapping of the left ventricle, observing two channels matching the fibrosis zone previously revealed by magnetic resonance imaging **(Figure 1)**. Subsequently, a hemodynamically unstable VT was induced with a 700-ms cycle (left bundle branch block, V4 transition, upper right axis). We positioned the Advisor™ HD Grid catheter in the area of interest, observing mid-diastolic signals on the inferobasal channel. We created ablation lines perpendicular to the diastolic channel **(Video 1)**.

In this case, the Advisor™ HD Grid multipolar catheter allowed us to perform substrate mapping with a high density of points and short mapping time. In addition, we were also able to map the VT in a very short time (approximately three minutes) due to poor hemodynamic tolerance, which required electrical cardioversion.

## Figures and Tables

**Figure 1:**
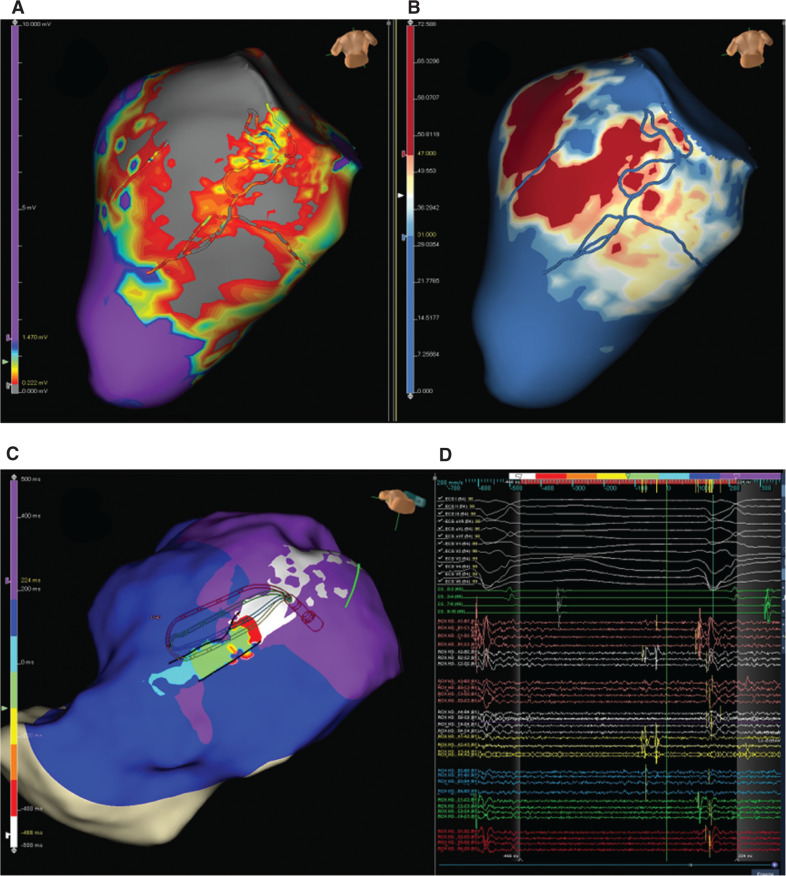
Substrate map in sinus rhythm. **A:** Two low-voltage channels can be seen in the inferobasal area of the left ventricle. **B:** ADAS three-dimensional map (ADAS3D Medical, Barcelona, Spain) and the channels visualized with magnetic resonance imaging. **C:** VT activation map. The VT isthmus is located in the channels seen in **A** and **B**. **D:** Diastolic signals seen in the A spline and the A2–B2 bipole of the HD Grid, located on the isthmus of the VT.

**Video 1.** Propagation map of a figure-of-eight reentrant circuit around the “protected isthmus” of the VT, delimited by two lines of block seen with black markers on the map. We can see the entrance signals of the VT in all the splines of the Advisor™ HD Grid.

